# Editorial: Predictive short/long-term efficacy biomarkers and resistance mechanisms of CAR-T immunotherapy treatment

**DOI:** 10.3389/fimmu.2023.1206441

**Published:** 2023-05-31

**Authors:** Jiali Cheng, Jin Jin, Liting Chen

**Affiliations:** ^1^ Department of Hematology, Tongji Hospital, Tongji Medical College of Huazhong University of Science and Technology, Wuhan, China; ^2^ Immunotherapy Research Center for Hematologic Diseases of Hubei Province, Wuhan, China

**Keywords:** chimeric antigen receptor-T cell therapy, efficacy, biomarkers, tumor characteristics, CAR-T cell characteristics, immune conditions

Chimeric antigen receptor (CAR)-T cell cells have exhibited impressive efficacy in hematological malignancies and have emerged as a potent therapy for a wider range of cancers (1–3). However, some patients are primarily resistant, and relapses after a period of remission are common. A clinical trial of anti-CD19 CAR-T therapy for acute B-cell leukemia (B-ALL) patients showed that 83% of patients achieved complete remission (CR), but without further consolidation, 65.4% of them relapsed (3). Identification of predictive short/long-term efficacy biomarkers and understanding the resistance mechanisms of CAR-T therapy are essential to avoid potential side effects for non-responders and initiate consolidation treatment for patients at high risk of relapse. Furthermore, these aims will contribute to the development of CAR-T therapy with superior potency.

The working processes of CAR-T cells include accessing the tumor site, recognizing target antigen positive tumor cells, and then attacking them (4). Generally, tumor factors, the immune status, and CAR-T-cell factors influence therapeutic outcomes and therefore are valuable in predicting the short/long-term prognosis. In this Research Topic, we collected original research articles, case reports, and reviews regarding recent advances in identifying predictors of CAR-T therapy response, and will be addressed according to whether they are assessed before or after CAR-T treatment below.

## Before therapy

### Patient baseline and tumor characteristics

As reviewed by Xu et al., the baseline characteristics of patients impact therapeutic outcomes. These include age, performance status, prior therapy exposure, and lactate dehydrogenase level, which were identified by retrospective studies of CAR-T clinical trials. These parameters are some of the simplest clinically derived parameters, are easy to evaluate, and can reflect the tumor burden and general condition of patients, which is of great significance in clinical practice. Interestingly,Gu et al. found that the sex of non-small cell lung cancer patients was associated with the expression level of PD-1 in T cells, affecting the response to immunotherapy. The impact of sexual hormones on the outcome of CAR-T-cell therapy in specific diseases remains to be investigated.

Besides, insights into tumor cells also inform the efficacy of CAR-T therapies. Target antigen expression and the genetic abnormalities are associated with the short/long-term efficacy of CAR-T therapy. As reported by Cai et al., epigenetic characteristics can serve as biomarkers for immunotherapy efficacy in hepatocellular carcinoma, possibly in concert with TP53 mutation, antitumor immune function, and drug resistance gene statuses. The role of epigenetic regulation in predicting the response to CAR-T therapy merits further investigation. Additionally, high-risk genetic features for traditional chemotherapy, such as P53 mutation/deletion, have a non-unified association with the outcomes of CAR-T therapy, warranting further exploration (2, 5, 6). Furthermore, the crucial role of antigen expression in CAR-T therapy has been well recognized (7, 8).

### CAR-T cell characteristics

The starting material (T cells) and the final products (CAR-T cells) of CAR-T therapy are known to influence treatment outcomes. Heterozygous mutation of *UNC13D* in T cells led to impaired *in vivo* expansion of CAR-T cells and inferior therapeutic efficacy (9). Studies of the correlations between T-cell functions and the efficacy of CAR-T therapy are important for screening patients of CAR-T therapy. The CAR design also affects outcomes. As reviewed by Kong et al., the incorporation of costimulatory domains improves the short/long-term survival. Furthermore, costimulatory signals vary greatly, with CD28 more likely to induce rapid but short-term proliferation and 4-1BB more likely to induce the opposite (10, 11). Feng et al. demonstrated that integration of the IL15/IL15 sushi structure into CAR-T cells improved their antitumor efficacy and the survival of tumor-bearing mice. It is important to evaluate the design of CAR under consideration of specific diseases. Furthermore, infusion product characteristics can affect response. A higher proportion of memory (CD45RO^-^CD27^+^CD8^+^) T cells favors longer-term remission (12). Strategies to increase the percentage of less differentiated memory-like CAR-T cells in infusion products will improve long-term disease control.

### Immune condition

In addition to the T-cell characteristics mentioned above, the tumor microenvironment (TME) is also an important part of immune status. More immune effector cells, such as CD8^+^ T cells, in the TME are associated with favorable tumor invasion of CAR-T cells and therefore better efficacy. Fibroblasts and the extracellular matrix hinder the infiltration of CAR-T cells, resulting in therapy resistance (13). Immunosuppressive cells, such as myeloid-derived suppressor cells and tumor-associated macrophages, inhibit the tumoricidal function of CAR-T cells, leading to an unsatisfactory response (14). Feng et al. developed anti-CD4 CAR-T cells to target T-cell malignancies and regulatory T-cell (with an immune suppression function) simultaneously to overcome suppressive immune conditions and enhance responses. Comprehensive evaluation of the impact of TME on therapy efficacy is significant but also challenging. Finding peripheral blood parameters that are representative of the immune status is clinically important.

## After therapy

### Monitoring tumors

The speed and depth of tumor remission have implications for long-term prognosis. Clearance of circulating tumor DNA (ctDNA) one week after CAR-T infusion occurred in 70% of patients who achieved persistent remission versus 14% of patients who experienced relapse. Patients with detectable ctDNA had a median event free-progression survival (FPS)/overall survival (OS) of 3/1.9 months, while the median FPS/OS was not reached for patients with undetectable ctDNA (15). Monitoring minimal residual disease at a higher resolution using readily available samples can help predict CAR-T therapy outcomes.

### Monitoring CAR-T cells

The pharmacokinetics and phenotype of CAR-T cells *in vivo* are important for predicting prognosis. A robust and sustainable response requires CAR-T cells to expand and persist. Patients with a higher peak CAR transgene level in peripheral blood are more likely to achieve long-term remission (16). By modeling the dynamics of CAR-T cells post-infusion, we can predict their proliferation capacity and identify factors related to their expansion (17). In B-ALL, rapid disappearance of CAR-T cells or recovery of B cells can inform impending relapse. The expression level of exhaustion genes in CD8^+^ T cells at 7 days post-infusion is related to response (18). De Matteis et al. reported a patient with a 100% senescent/exhausted phenotype of CD8^+^ CAR-T cells who relapsed and failed to respond to anti-PD1 therapy, supporting the value of monitoring the functional status of CAR-T cells in predicting efficacy.

### Monitoring cytokines/chemokines

Cytokines/chemokines reflect the interactions between the immune system and tumors and are regulators of these interaction. The level of MCP-1 before lymphodepletion, IL-7, and MIP3α at the peak of cytokine release syndrome are independent protective factors for predicting the long-term survival of B-NHL patients receiving CAR-T therapy (19). The concentration of IL-15 was found to be related to CAR-T cell expansion and lymphoma remission (20). In summary, cytokines/chemokines are feasible biomarkers that can be used to predict the outcome of CAR-T cell therapy.

## Conclusion

Altogether, as more clinical trials have been performed and analytic techniques have advanced, additional factors associated with tumors, CAR-T cells, and the immune status have been identified to predict CAR-T therapy responsiveness. Comprehensive consideration of the roles of these factors in determining therapeutic outcomes is required. More efforts are needed to overcome the barriers of resistance and relapse.

## Author contributions

LC supervised the whole process. JCand JJ wrote the manuscript. All authors approved it for publication.
